# A case report of heterotopic gastric mucosa in the rectum treated by endoscopic submucosal dissection and a systematic review

**DOI:** 10.1097/MD.0000000000034491

**Published:** 2023-07-28

**Authors:** Dunhuang Peng, Ting Qiu, Shaohua Chen, Weitao Hu, Taiyong Fang

**Affiliations:** a Department of Gastroenterology, The Second Affiliated Hospital of Fujian Medical University, Quanzhou, Fujian, P.R. China; b Department of Pathology, The Second Affiliated Hospital of Fujian Medical University, Quanzhou, Fujian, P.R. China.

**Keywords:** endoscopic submucosal dissection, heterotopic gastric mucosa, literature review, rectum

## Abstract

Heterotopic gastric mucosa (HGM) can occur in all segments of the gastrointestinal tract, but rectal is very rare. In recent years, rectal HGM is more often treated by endoscopic resection (ER).

**Patient concerns:**

A 28-year-old female was admitted to the hospital with the chief complaint of “a rectal lesion found on physical examination”.

**Diagnoses:**

Heterotopic gastric mucosa (HGM).

**Interventions:**

An endoscopic submucosal dissection (ESD) was performed to completely dissect the lesion.

**Outcomes:**

The patient recovered well at 1 month of follow-up and did not suffer from further blood in the stool.

**Lessons:**

Rectal HGM has acid secretion function and HP can be colonized, causing a variety of symptoms such as abdominal pain, bloody stool, and anal pain and has the potential risk of malignant transformation; resection is the best treatment method, and ESD has its unique advantages and can be promoted in the clinic.

## 1. Introduction

Heterotopic gastric mucosa (HGM) is the most reported epithelial ectopic, a morphologically normal tissue displaced from a foreign anatomical site in which the gastric mucosal tissue is clearly distinguished from the surrounding mucosa and completely separated from its organ of origin, and can occur in all segments of the digestive tract. HGM tends to occur in the esophageal segment and presents as a flat or superficial elevated lesion shaped like the entrance to the esophagus. Patchy ectopic gastric mucosa can be seen in the upper esophagus in nearly 18% to 30% of the population,^[1]^ while the detection rate in the duodenum and small intestine is about 0.1% to 10%,^[2,3]^ and in the rectum is very rare. Rectal HGM endoscopically appears as flat or superficial elevated lesions shaped like the entrance to the esophagus. Recently, the treatment of rectal HGM is more likely to be endoscopic resection (ER) rather than the surgical procedure in the past. The current study retrospectively analyzed the clinical manifestations and histopathological features of rectal HGM.

## 2. Case report

**Figure 1.**
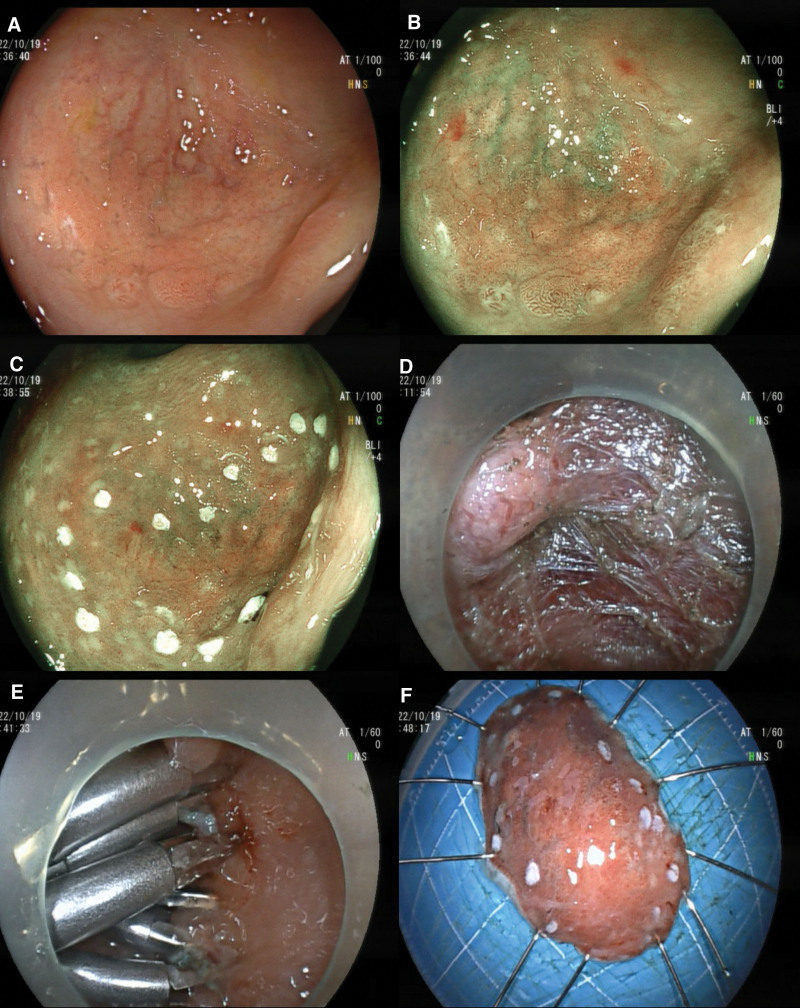
Heterotopic gastric mucosal ESD treatment of low rectum. (A) White-light; (B) BLI electronic staining; (C) Lesion markers; (D) Peeling along the submucosa; (E) Wound closure; (F) Specimen fixation 15mm*25mm. ESD = endoscopic submucosal dissection.

**Figure 2.**
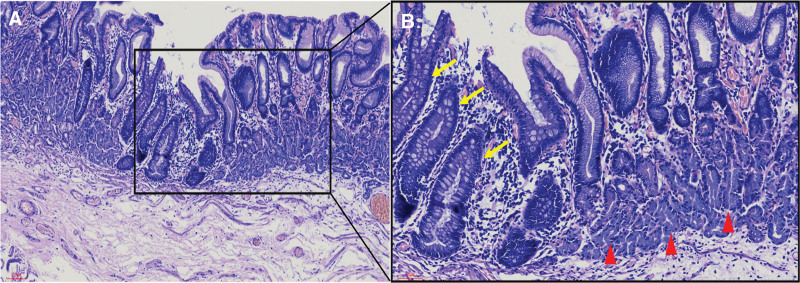
Histology (hematoxylin & eosin staining) of rectal fundic gland mucosa. (A) Rectal mucosa and fundic gland tissue (magnification ×10); (B) Gastric fundic glands (red triangle) and rectal glands (yellow arrows) (magnification ×20).

A 28-year-old female was admitted to the hospital with the chief complaint of “a rectal lesion found on physical examination.” The physical examination revealed a flat lesion in the rectum about 3 cm from the dentate line, 0-IIb in size, about 15*20 mm, with a smooth surface and visible borders. This patient was in good health and denied any history of bloody stools, anal fissures, or fistulas. It was difficult to ensure negative margins by endoscopic mucosal resection (EMR), considering that the lesion was flat and slightly depressed locally, so an endoscopic submucosal dissection (ESD) was performed to completely dissect the lesion (Fig. 1). Postoperative pathological histology suggested that fundic adenoid tissue was visible under the normal glands of the rectal mucosal layer (Fig. 2), and the horizontal and vertical cut edges were not observed. Therefore, the diagnosis of HGM was given. The patient recovered fine at 1 month of follow-up and did not suffer from further blood in the stool.

## 3. Methods

Pubmed, MEDLINE, EMBASE, and Google Scholar were utilized to search the medical literature from 1939 (initial description of rectal HGM)^[4]^ to October 2022. All relevant papers were available and evaluated with the words “heterotopic gastric mucosa,” “gastric heterotopic,” and “rectum” as search terms, without restriction on language. In the case of multiple publications from the same author, each case was identified according to its demographic characteristics, and duplication was avoided. For HGM patients, the following data were extracted: age and sex, size, morphology, location, histological features, clinical symptoms, presence of associated deformities/complications, treatment modality and recurrence rate, and symptom relief rate. Based on the Paris classification,^[4]^ HGM was phenotyped morphologically into elevated lesions (0-I), flat lesions (0-II), and depressed lesions (0-III). Type 0-I was further divided into tipped (0-Ip) and untipped (0-Is). Type 0-II was classified into 3 subtypes, 0-IIa, 0-IIb, and 0-IIc corresponding to slightly elevated, flat, and slightly depressed lesions, respectively. The cutoff between type 0-I and type 0-IIa was the height of augmentation up to 2.5 mm (thickness of biopsy forceps closing), and the boundary between type 0-III and type 0-IIc was the depth of depression up to 1.2 mm (thickness of individual forceps of biopsy forceps opening). Lesions with both slight elevation and slight depression were classified into 0-IIc + IIa and 0-IIa + IIc types by the elevation/depression ratio. Lesions with a combination of depression and slight depression were classified as III + IIc and 0-IIc + III types according to the ratio of depression/slight depression.

For comparisons between measurement data, the *t* test was applied; for count data, expressed as rates (%), comparisons were performed by chi-square test, and if T < 5, the Fisher exact probability test was conducted. Two-tailed P values < 0.05 were considered statistically significant. All statistical analyses and calculations were performed by the IBM SPSS statistics 25 software. This study was approved by the Clinical Research Ethics Committee of the Second Affiliated Hospital of Fujian Medical University ([2022]251). The patient has given consent for the case to be published.

## 4. Results

Since the first description by Ewell and Jackson in 1939,^[5]^ 47 cases of rectal HGM have been reported, including this 1 case in the present study. 9 studies were excluded: 3 were duplicate cases, and 6 were non-rectal HGM cases or with incomplete information. A total of 47 cases were evaluated (Table 1). The median age of the patients was 25 years, ranging from 1 day after birth to 69 years, with 18 minors (<18 years) and 29 adults (≥18 years). A total of 25 patients (53.2%) were males.

**Table 1:** The statistics table of rectal heterotopic gastric mucosa cases.

Age (yr)/Sex	Malformation	Location	Morphology	Size (mm)	Complication	Mucosa type	Treatment
0, F	Multiple	Proximal	0-Ib		No	Mixed	Med
1, F	No	Low	0-Is	25	No	Oxyntic	Surgery
2, F	No	Low	0-IIa	30	No		Surgery
3, F	No	low	0-IIa + IIc	30	Bleeding	Oxyntic	ESD
3, M	No	Low	0-I		No	Oxyntic	Surgery
4, M	No	Low	0-IIa + IIc	50	Ulcer		Surgery
4, F	RD	Low	0-II	35	No	Oxyntic	Med
4, M	No	Proximal	0-IIa	40	Bleeding	Oxyntic	EMR
4, M	No	Low	0-Is	15	Ulcer	Oxyntic	EMR
5, M	No	Low	0-Is	10	No	Oxyntic	Surgery
5, F	No	Low	0-IIa + IIc	40	No	Oxyntic	EMR
5, F	RD	Proximal	0-III	20	Perforation	Oxyntic	Med
6, M	No	Low	0-I	1	Ulcer	Oxyntic	Med
7, M	No	Middle	0-Is	15	No	Oxyntic	EMR
10, M	RD	Proximal	0-IIa	20	Ulcer	Oxyntic	Surgery
10, M	No	Proximal	0-Is	30	Ulcer	Oxyntic	Surgery
14, F	RD	Low	0-IIa	50	No	Oxyntic	ESD
16, M	No	Low	0-I	30	Ulcer	Oxyntic	Surgery
19, F	Multiple	Low	0-Ip		Perforation	Oxyntic	Med
20, M	No	Proximal	0-III	15	Ulcer	Oxyntic	EMR
22, M	No	Low	0-II	50	No	Oxyntic	Med
22, M	No	Middle	0-IIa + IIc	40	No	Oxyntic	Surgery
25, M	No	Middle	0-IIa	40	Bleeding	Oxyntic	EMR
26, M	RD	Middle	0-Is	60	No	Oxyntic	Surgery
28, F	No	Low	0-I		No	Mixed	Med
28, F	No	low	0-IIb	20	Bleeding	Oxyntic	ESD
31, M	No	Middle	0-IIa	20	No	Oxyntic	Med
33, M	No	Low	0-Ip	18	Anal pain	Oxyntic	EMR
34, M	No	Low	0-Is	25	No	Oxyntic	EMR
35, F	RD	Middle	0-IIb	10	No	Oxyntic	Ablation
36, F	No	Low	0-Is	25	No	Oxyntic	EMR
45, M	No	Proximal	0-Ip	20	No	Mixed	Surgery
45, M	Diverticula	Low	0-IIa + III	30	Bleeding	Oxyntic	ESD
46, F	No	Proximal	0-Ip	15	No	Oxyntic	Surgery
46, F	Rd	Proximal	0-IIb	15	No	Oxyntic	Med
46, M	No	Proximal	0-IIb	30	No	Cardiac	Med
46, M	No	Middle	0-IIc	15	Ulcer	Oxyntic	EMR
47, F	No	Proximal	0-Ia	30	No	Oxyntic	Ablation
48, F	RD	Low	0-IIb	30	No	Oxyntic	Surgery
48, F	RD	low	0-IIc	30	Bleeding	Oxyntic	Surgery
51, M	No	Proximal	0-I	30	No	Mixed	Med
51, F	Diverticula	Low	0-IIa + IIc	20	No	Mixed	EMR
51, M	No	Low	0-Ia	40	No	Oxyntic	Med
51, M	No	Low	0-Ib	1	No	Antral	Surgery
52, F	No	Low	O-IIa + III	12	Anal pain	Oxyntic	EMR
58, F	No	Low	0-Ila			Oxyntic	Med
63, F	No	Low	0-IIb	30	No	Oxyntic	ESD

EMR = endoscopic mucosal resection, ESD = endoscopic submucosal dissection, F = female, Hp = Helicobacter pylori, M = male, med = conservative treatment, RD = rectal duplication.

## 5. Pathologic features

### 5.1. Localization

The rectum was divided into 3 segments, 27 cases were located in the low rectum (57.4%), 8 cases (17.0%) in the middle rectum, and 12 cases (25.5%) in the proximal rectum.

### 5.2. Endoscopic morphology

The mean lesion size was 27.1 ± 12.7 mm, varying from 1 mm to 60 mm. The morphology of HGM was typed according to the Paris classification.^[4]^ There were 24 cases (51.1%) of flat lesions (type 0-II), including 8 cases (17.0%) of slightly elevated lesions (type 0-IIa), 7 cases (14.9%) of flat lesions without elevation (type 0-IIb), 6 cases (12.8%) of superficial elevation with depression (type 0-IIa-IIc), and 3 cases without further typing. In addition, there were 19 cases (40.4%) with polypoid elevations (0-Is, 0-Ip, and 0-Isp types) and 4 cases (8.5%) with deep depressions (0-III types), which included the ulcerated type. The proportion of type I was more in minors than in adults, with no significant difference (Table 2). The presence of diverticula or repetitive digestive tract malformations was found in 11 cases (23.4%). Other complications were observed in 10 cases (21.3%): 8 cases (17.0%) of rectal mucosal ulcers and 2 cases of rectal perforation (4.3%).

**Table 2:** Characteristics of ectopic gastric mucosa based on age.

Age (yr)	Size (mm)	*P*#	Pari	*P*##	Symptoms	P*	Treatment	P**	Treatment	P***
			I/II/III (I%)		(anal-rectal %)		(Surgery%)		(ESD%)	
<18	29.3	.83	9/8/1 (50)	.73	13/18 (72.2)	.02	8/18 (44.4)	.15	2/18 (11.1)	.14
≥18	25.8		12/14/3 (40)		11/29 (37.9)		7/29 (24.1)		3/29 (10.3)	

ESD = endoscopic submucosal dissection

*P*# = comparison of diameters between the 2 groups

*P*## = comparison of the proportion of Type I

P* = comparison of the percentage of anal pain

P** = comparison of the percentage of surgery

P*** = comparison of the percentage of ESD; *P* < .05 was statistically significant.

### 5.3. Histology

The histological type of HGM was reported in 44 cases (93.6%) (Table 1), with the fundic gland mucosa being the most common in 37 cases (78.7%); mixed mucosa of the pyloric and fundic glands in 5 cases (10.6%), mucosa of the pyloric gland in 1 case (2.1%), and mucosa of the cardia gland in 1 case (2.1%). Additionally, there was 1 case of intestinal metaplasia, 1 case of pancreatic adenoma with low-grade intraepithelial neoplasia, and 1 case with high-grade intraepithelial neoplasia. The detection of HP by Giemsa or Warthin Starry staining revealed positive results in 2 out of 11 cases (18.2%). And in 1 case, HP was present in the HGM of the rectum but not in the stomach.^[6]^

### 5.4. Clinical features

Clinical symptoms are more likely to be abnormal bowel movements, abdominal pain, blood in the stool (including blood in the stool, blood dripping from the stool, blood on the paper, etc), and anal pain, etc, with varying durations. 11 patients were asymptomatic (23.4%). In this group, 3 patients had positive fecal occult blood tests and/or were accompanied by iron-deficiency anemia. 5 patients (10.6%) had abdominal symptoms. Anorectal-related symptoms were present in 24 patients (58.5%), and anal pain and blood in the stool were more common in children (<18 years), occupying 13/18 (72.2%), which was significantly higher than the percentage of 11/29 (37.9%) in adults, with statistically significant differences (Table 2). Two girls aged 5 years and 19 years presented with abdominal pain and rectal perforation.

### 5.5. Treatment

32 cases (68.1%) were treated with resection, including 15 (31.9%) surgical resections, 12 (25.5%) EMR resections, and 5 (10.6%) ESD resections. The proportion of the minor group receiving surgical treatment (44.4%) was higher than that of adults (24.1%), with no significant difference. Of the cases, 20 (42.6%) received pharmacological treatment, both conservative and preoperative and postoperative, including H2 receptor antagonists, proton pump inhibitors, bismuth and antibiotic therapy, and/or eradication of HP. No significant bleeding or perforation complications were observed in any of the 5 included ESD-treated cases. The conservative treatment was effective in all cases in terms of symptom control and ulcer healing. After HGM resection, with CT in all cases and colonoscopy in 21 cases during a median follow-up of 21 months (range 1–84), no recurrence was observed in any case.

## 6. Discussion

Regarding the pathogenesis of HGM, Morrison^[7]^ suggested that it is related to incorrect differentiation of endodermal pluripotent stem cells during embryonic development, and Wolff^[8]^ further divided it into 2 categories: if more than one type of epithelium is involved in the process or if the whole layer of ectopic mucosa consists of gastric fundic mucosa, it is considered to be caused by congenital developmental abnormalities; if the tissue is exclusively pyloric gland epithelium or only dispersed principal and mural cells, it is considered to be acquired. The lesions in the present case are consistent with congenital developmental abnormalities. Intestinal epithelial metaplasia of the gastric mucosa is mostly acquired, caused by local injury or inflammation, and is an adaptive response of cells from primitive anatomical sites to injury and/or inflammation, like Barrett esophagus.^[9]^ It is certainly possible that HGM is the result of a combination of factors. The HGM is commonly combined with gastrointestinal abnormalities, such as gastrointestinal duplication and diverticula. HGM tends to be seen at the edge of the diverticula or inside the diverticula, and some of them show morphology similar to the diverticula.^[10]^ There is no evidence to clarify whether gastrointestinal malformations and HGM occur simultaneously or sequentially. Nevertheless, their coexistence will lead to frequent inflammatory stimulation of HGM, resulting in an increased incidence of bleeding, perforation, and heterogeneous hyperplasia, which needs to be concerned. It is helpful to determine the clinical relevance and prognosis through a systematic review of rectal HGM cases and a clinicopathological classification of HGM. Among minors, polypoid bulges (type I) are more common than in adults which may be due to fewer endoscopies in children. And among adults, flat types (type II) are more common. Most flat lesions are found incidentally during colonoscopy in asymptomatic patients. Since the diagnosis of HGM cannot be confirmed by the naked eye and pathological histology is the gold standard, it suggests that we should pay attention to colonoscopy and take a biopsy or remove the lesion for examination in time if there is a suspicious lesion. 99mTc scan can show the uptake point of the ectopic gastric mucosa. Some cases reported multiple ectopic gastric mucosae located in different parts of the gastrointestinal tract,^[11,12]^ for lesions in the small intestine, which are difficult to cover by conventional gastroscopy and colonoscopy, 99mTc tracing shows some degree of diagnostic value. Notably, a recent systematic review^[13]^ reported that in 9 cases of 99mTc scans, the presence of ectopic gastric mucosa in the rectum was found in 7 cases, but was not confirmed endoscopically in one of them; in 2 patients without uptake sites, HGM was detected endoscopically. In another study,^[14]^ 99mTc scans of 9 children with rectal HGM showed only 3 cases (33%) with rectal radionuclide accumulation. To some extent, it is controversial whether the 99mTc scan is recommended for all patients.

Although rectal HGM is very rare, it should be considered in the differential diagnosis of rectal lesions or related symptoms due to its presence of acid secretion and possible co-infection with Helicobacter pylori (HP). Galan et al first demonstrated the acid secretion capacity of HGM in the absence of any stimulation by performing a 24-hour esophageal PH assay.^[15]^ In 5 cases of ulcerated HGM, the ulcers healed after acid suppression treatment, indirectly proving the presence of acid secretion in HGM. Hydrochloric acid and gastrin secreted by HGM can corrode and stimulate the walls of the digestive tract. Rectal HGM may be associated with abdominal pain and blood in the stool, and these symptoms can be improved by acid suppression therapy, providing indirect evidence for the acid-secreting function of HGM.^[10]^ It was discovered by Alagozlu et al^[16]^ that HP can colonize the ectopic gastric mucosa. Dye et al^[17]^ reported that the successful eradication of HP from rectal HGM resulted in the relief of abdominal pain in patients. Therefore, HP eradication treatment is also feasible for patients with symptomatic rectal HGM with co-infection of HP. Two cases of HGM with intestinal metaplasia and intraepithelial neoplasia were diagnosed incidentally during colonoscopy in the asymptomatic population.^[18,19]^ Studies in animals have revealed that HGM can be carcinogenic,^[20]^ as well as in the human esophagus, small intestine, gallbladder, and colon.^[14]^ A case of rectal HGM with high-grade intraepithelial neoplasia^[21]^ suggests that HGM is at risk of malignancy. These results which were included suggest that long-standing HGM of the rectum may be at some risk of cancer and should be followed endoscopically or resected if available. Conventional EMR has certain limitations, for lesions over 20 mm flat type cannot be completely snapped, often need to be resected in pieces, which is not conducive to the judgment of the incision margin, and the recurrence rate is found to be higher than EMR and ESD in endoscopic piecemeal mucosal resection cases of lateralized tumors; Surgical procedures are more invasive and require removal of part of the intestinal segment; ESD allows peeling through the submucosal space, preserving the integrity of the intrinsic muscular layer of the intestinal wall and enabling complete excision of the lesion, allowing removal of flat lesions exceeding 20 mm. In the present case, the lesion reached 20 mm and was morphologically non-polypoidal bulge with a Paris classification of type 0-IIb, and the entire lesion was slightly depressed. The EMR treatment trap tended to slide over the lesion and could not be fixed, whereas ESD could effectively address these limitations and remove the lesion completely, avoiding unnecessary surgery.^[22]^ It is considered that although ESD is more difficult and risky to operate than EMR, for the rectum, compared with the right hemicolon and sigmoid colon, the distance to the anus is closer, the endoscope is not twisted, easy to enter and exit, free to rotate, and less difficult to operate. None of the included ESD treatment cases showed significant bleeding and perforation even if the lesion reached 50 mm, which is considered to be related to this factor; and the low rectum belongs to the extraperitoneal site, so even if it is perforated, the infection is relatively limited. ESD has been increasingly used in the treatment of rectal HGM in recent years since its first report in 2016.^[10,13,14,23]^

It is expected that more cases and studies will be available to assess the predicted risk of HGM malignancy, and treatment modalities. In comparison with EMR, ESD allows better pathological evaluation, reduces lesion residuals and recurrence rates, and the prognosis of all cases followed is good. The ESD resection of rectal HGM has its unique advantages and can be promoted in the clinic.

## Author contributions

**Investigation:** Shaohua Chen.

**Methodology:** Shaohua Chen.

**Validation:** Ting Qiu, Shaohua Chen, Weitao Hu, Taiyong Fang.

**Writing – original draft:** Dunhuang Peng, Taiyong Fang.

**Writing – review & editing:** Dunhuang Peng, Weitao Hu, Taiyong Fang.
